# Tobacco Product Waste: An Environmental Approach to Reduce Tobacco Consumption

**DOI:** 10.1007/s40572-014-0016-x

**Published:** 2014-05-06

**Authors:** Thomas E. Novotny, Elli Slaughter

**Affiliations:** Graduate School of Public Health, San Diego State University, 5500 Campanile Drive, Hardy Tower 119, San Diego, CA 92182 USA

**Keywords:** Tobacco product waste, Cigarette filters, Extended producer responsibility, Tobacco consumption

## Abstract

Cigarette butts and other tobacco product wastes (TPW) are the most common items picked up in urban and beach cleanups worldwide. TPW contains all the toxins, nicotine, and carcinogens found in tobacco products, along with the plastic nonbiodegradable filter attached to almost all cigarettes sold in the United States and in most countries worldwide. Toxicity studies suggest that compounds leached from cigarette butts in salt and fresh water are toxic to aquatic micro-organisms and test fish. Toxic chemicals have also been identified in roadside TPW. With as much as two-thirds of all smoked cigarettes (numbering in the trillions globally) being discarded into the environment each year, it is critical to consider the potential toxicity and remediation of these waste products. This article reviews reports on the toxicity of TPW and recommends several policy approaches to mitigation of this ubiquitous environmental blight.

## Introduction

Cigarette butts and other tobacco product waste (TPW) items are the most ubiquitous form of litter worldwide, with an estimated 4.5 trillion of the estimated annual 6 trillion globally consumed cigarettes deposited as butts somewhere into the environment each year [1]. This material comprises the largest percentage of waste (approximately 19 %–38 % of total waste products by count) collected globally during the coastal cleanups each year (See Ocean Conservancy Data for 2012, [Table 1]). At a local level, data from a City of San Francisco Street Litter Audit revealed that 24.6 % by count of all litter items collected were from tobacco products (including butts, wrappers, and packages) [2].

**Table 1:** Top 10 marine debris items collected, international coastal cleanup

Rank	Debris item	Number of debris items	Percentage of total debris items
1	Cigarettes/cigarette filters	2,117,931	19 %
2	Food wrappers/containers	1,140,222	10 %
3	Beverage bottles (plastic)	1,065,171	10 %
4	Bags (plastic)	1,019,902	9 %
5	Caps, lids	958,893	9 %
6	Cups, plates, forks, knives, spoons	692,767	6 %
7	Straws, stirrers	611,048	6 %
8	Beverage bottles (glass)	521,730	5 %
9	Beverage cans	339,875	3 %
10	Bags (paper)	298,332	3 %
	Top 10 total debris items collected	8,765,871	80 %
	Total debris items collected worldwide	10,957,338	100 %

Source: Ocean Conservancy, 2012: http://www.oceanconservancy.org/our-work/international-coastal-cleanup/top-10-items-found-1.html.

Although it is difficult to estimate what percentage of the trillions of cigarettes consumed globally each year are discarded as waste, bans on indoor smoking may have exacerbated the accumulation of TPW outdoors. Residents, business owners, and politicians have reported an increase in the quantity of cigarette butts littered after bans on indoor smoking took effect in local areas [3–5]. In the United Kingdom, a report by the advocacy group Keep Britain Tidy [6], estimated a 43 % increase in the number of littered cigarettes attributable to a ban on indoor smoking. Keep Britain Tidy is supported by the tobacco industry, which in the past has used these data as an argument to undermine clean indoor air laws [7]. One community (Tacoma, Washington, USA) [8] conducted a litter study in 2010 and estimated that 1 in 3 smoked cigarettes are discarded into the environment. The American Legacy Foundation surveyed a national sample of 1000 smokers and found that most (74.1 %) admitted disposing of butts on the ground or out of a car window at least once in their lives [9]. Recent observational studies of smokers document that a majority (76.7 %; 95 % CI 70.8–82.0 %) of 219 subjects littered their cigarette butts; this behavior appears to be the norm among smokers in urban settings, even in the presence of appropriate waste receptacles [10].

**Fig. 1:**
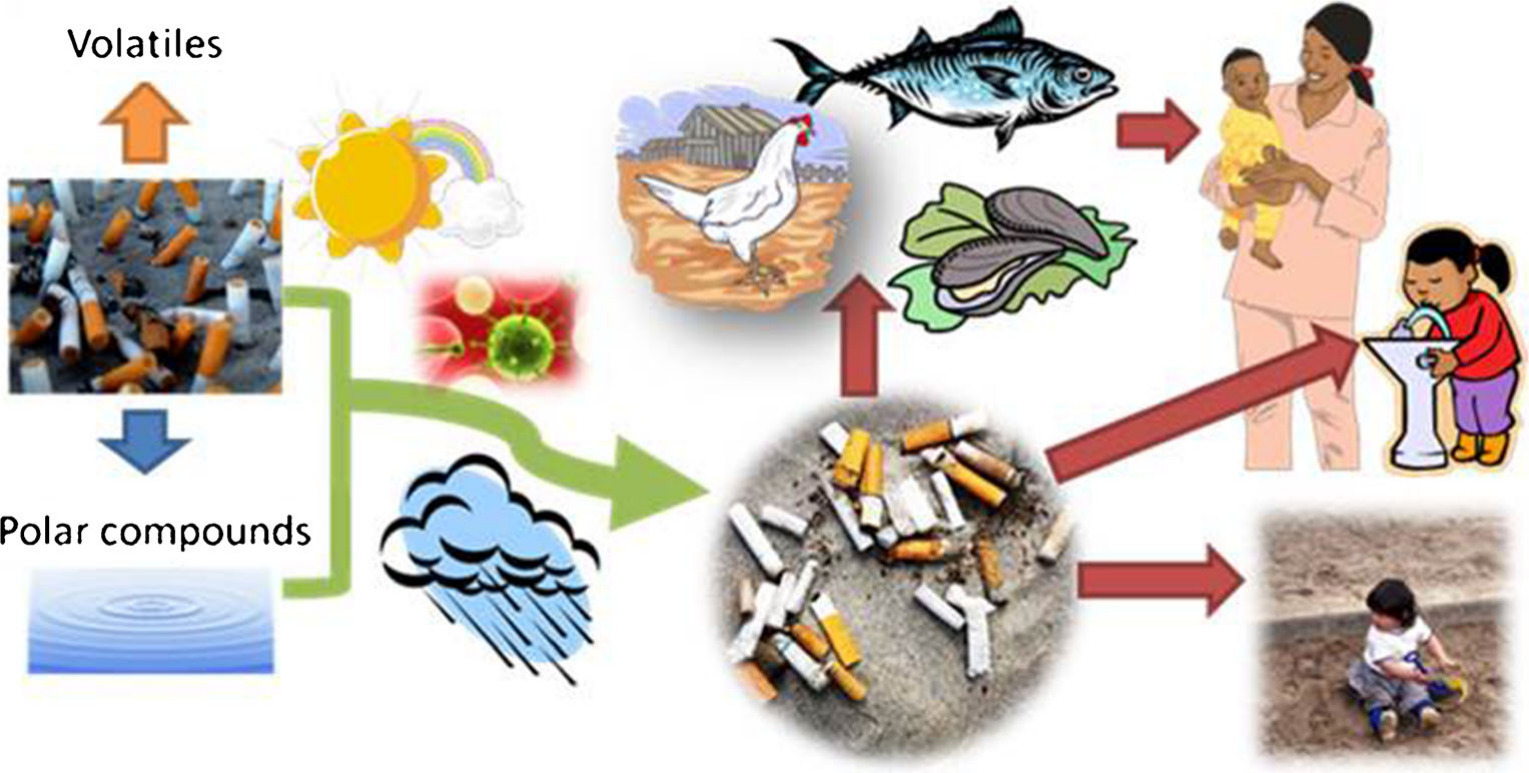
Possible pathways for human health risks due to TPW

Given that the weight of 20 cigarette filters is 0.12 ounces (3.4 gm) [11], the estimated discarded waste from U.S. cigarette consumption in 2011 alone (292.8 billion) [12] would weigh about 49.8 million kg; this estimate does not include the weight of remnant tobacco, discarded packages, lighters, matches, and other tobacco products such as cigars, e-cigarettes, and smokeless tobacco. The casual disposal of TPW is a normative part of smoking and creates a potentially toxic environmental burden and potentially a risk to human health through environmental contamination (Fig. 1). TPW is washed by the rain or by street cleaning from urban sidewalks and streets into the storm drains and then into the larger aquatic environment [13]. This review will evaluate the potential for environmental toxicity due to chemicals leached out of the main TPW element (cigarette butts); we will propose policy options for mitigating TPW.

## Potential Toxicity of Tobacco Product Waste

TPW is unlikely to be thought of as a toxic waste product by smokers, nonsmokers, manufacturers, or communities. Further, it has not yet been considered as such by state or local environmental protection agencies. Nonetheless, the numerous chemicals found in cigarette tobacco and generated when the tobacco burns [14•] are likely to be harmful to the environment, including pesticides, herbicides, insecticides, fungicides, and rodenticides that are used in the agricultural production of tobacco products [15]. In fact, many of the chemicals found in tobacco products are included in the Environmental Protection Agency’s Toxic Release Inventory (TRI) Program [16]. Chemicals covered by the TRI are those that cause 1 or more of the following: cancer or other chronic human health effects, significant adverse acute human health effects, or significant adverse environmental effects. Tobacco contains nicotine (which is a chemical also used in plant pesticides), polyaromatic hydrocarbons, various carcinogenic nitrosamines, ammonia, acetaldehyde, formaldehyde, phenol, pyridines, acetone, and heavy metals, among other toxicants [17••, 18•]. We will next review the evidence that these chemicals may adversely impact the environment.

Agricultural chemicals have been found to be present in cigarette smoke. For example, Dane et al. [19] found 3 previously undetected pesticides (flumetralin, pendimethalin, and trifluralin) in both mainstream and side stream cigarette smoke. Cigarette filters are theoretically designed to absorb various constituents of cigarette smoke, including gaseous emissions and particulates, and, thus, if harmful chemicals in tobacco leaf are transferred to cigarette smoke, they could also be retained by cigarette filters and tobacco remnants in discarded butts. Pesticides are manufactured to effectively kill target organisms at relatively low doses. If these chemicals leach from discarded cigarette filters, they could potentially be toxic in various environments and could bio-accumulate in the human food chain.

Ethyl phenol is used in the tobacco industry as a flavoring agent and is present in cigarette smoke [20]. It bioconcentrates in aquatic organisms [21]. Thompson et al. [22] identified a relatively high Lethal Concentration (LC) 50 (the concentration at which there is 50 % lethality in a bioassay) for ethyl phenol at 150 mg/L. Although the concentration of ethyl phenol in mainstream smoke of a single cigarette is less than the LC50, it may still represent a potential toxicant in TPW. This toxicity might occur because cellulose acetate, the major component of cigarette filters, has been shown to effectively remove phenols from cigarette smoke [23–26]. Consequently, ethyl phenol may be present in the discarded cigarette filter at much higher concentrations than in cigarette smoke and may leach into the environment.

Approximately 0.6 % to 3.0 % of the dry weight of tobacco is nicotine, which has been used as a plant pesticide since the 15th Century [27]. It became a popular pesticide in the United States in the 1940s and 50s [28], but nicotine-based pesticides have not been sold in the United States since 2008 [29]. Nicotine is known to be acutely toxic to animals and humans [30, 31]. An average cigarette yields approximately 1–2.3 mg of nicotine [32, 33] and, in this low concentration, nicotine acts as a stimulant; it is the main chemical responsible for tobacco dependence. Of note is that the nicotine content in cigarettes increased 1.6 % between 1998 and 2005 [34].

Additives are reported to constitute 10 % of the weight of the tobacco in a cigarette and 4 % of the total weight of the cigarette [11]. Additives (such as menthol) make cigarette smoke more palatable and appealing to the consumer, especially those who are initially experimenting with smoking. Humectants, for example, increase shelf life, and along with sugars, aid in the dissolution of nicotine, making smoke milder and easier to inhale. Diethylene glycol, commonly used as automotive antifreeze, was added to cigarette tobacco as a humectant in the 1930s [15] and removed as a result of public advocacy in the 1980s. This sequence of events, however, contradicts the usual expectation for consumer products such that safety is established for their content before a product is used. (Interestingly, history is repeating itself now with the increasing popularity of e-cigarettes, which produce several vaporized chemicals, have no regulatory oversight, and contain varying amounts of nicotine.)

Cigarette smoke is a complex mixture of gases and submicron-size particulate matter [35]. Cigarette tar, technically the material deposited on a filter when the smoke is passed through, is used as a catch-all term for the particulate components of cigarette smoke, except for alkaloid compounds such as nicotine [36]. Tar is comprised of organic and inorganic compounds, many of which are carcinogenic [35]. The discarded cigarette filter may retain many of these potential carcinogens that may be leached into the environment and transferred to aquatic organisms, some in the human food chain.

Few studies have addressed the toxic effects of TPW on living things, but aquatic ecosystems, such as shorelines and waterways, may be the most vulnerable settings, as the majority of land-based litter is ultimately deposited into these environments [17••].

### Evidence on Environmental Toxicity Due to Tobacco Product Waste

Several studies have shown chemicals that leach from cigarette butts can be acutely toxic to aquatic organisms [11, 37, 38]. Moriwaki et al. [39] found that arsenic, nicotine, PAHs, and heavy metals such as cadmium and lead are released into the environment as part of roadside TPW. In this study, roadside waste was collected in a Japanese suburb prospectively over a 4-month period. The distribution, quantity, and types of waste were studied, as well as the environmental loading of PAHs and other pollutants over time from this waste. Environmental contamination by heavy metals, such as lead, copper, chromium, and cadmium, as well as by PAHs (Table 2) from cigarette butt waste, was confirmed.

**Table 2:** Polyaromatic hydrocarbons (PAHs) in roadside cigarette butt waste and roadside soil, Japan, 2009

PAHs	Concentration (mg/kg wet)		Load potential (mg/km/month)^1^
	Cigarette butts	Roadside soil	
Fluorene	0.028	0.01	0.0023
Phenanthrene	0.078	0.14	0.0063
Anthracene	0.071	0.0058	0.00057
Pyrene	0.091	0.36	0.0074
Benzo(*a*)anthracene	0.026	0.084	0.0021
Chrysene	0.044	0.11	0.0035
Benzo(*b*)fluoranthene	0.031	0.088	0.0025
Benzo(*k*)fluoranthene	0.015	0.055	0.0012
Benzo(*a*)pyrene	0.031	0.12	0.0025
Dibenzo(*a,h*)anthracene	0.0065	0.016	0.00053
Benzo(*g,h,i*)perylene	0.031	0.093	0.0025
Total	0.39	1.1	0.032

^1^Values of load potential were calculated using the quantity of cigarette butts per month, concentration of PAHs, and length of sampling environment (3.2 km).

Reprinted with permission from Waste Management. Vol 29(3). Moriwaki H, Kitajima S, Katahira K. Waste on the roadside, ‘poi-sute’ waste: its distribution and elution potential of pollutants into environment. p. 1192–7. Copyright 2009, with permission from Elsevier. [39].

Moerman and Potts determined the concentration of Al, Ba, Cd, Cr, Cu, Fe, Mn, Ni, Pb, Sr, Ti, and Zn from cigarette butts in aqueous solution, including assessment of pH effects and soaking time on metal concentration leached [18•]. All metals were detected in leachates 24 hours after cigarette butt addition, with the exception of Cd, and were released at varying rates. This research suggests that cigarette butts are potential sources of heavy metal environmental contamination and have the potential to cause acute and chronic harm to various organisms.

Register [11] followed the USEPA’s 1996 “Aquatic Invertebrate Acute Toxicity Test, Freshwater Daphnids” protocol in performing toxicity bioassays of cigarette butts. Cigarette butt leachate was prepared by allowing cigarette butts to soak in deionized water for 1 hour. This study found that leachates from smoked cigarette tobacco, smoked cigarette filters, and unsmoked cigarette filters were acutely toxic to the freshwater cladoceran Daphnia magna at 0.125 and 0.25, 1, and 2, and greater than 16 cigarette butts/L (LC50), respectively. This test took place over a 48-hour period, and survival was the single endpoint.

Warne et al. [38] prepared cigarette butt leachate by placing cigarette butts in water and shaking for 1 hour. The LC50 of leachates from smoked cigarette butts, smoked cigarette filters, and unsmoked cigarette tobacco were reported for the freshwater cladoceran Ceriodaphnia dubia at 0.05, 0.15, and 1.7 cigarette butts/L, respectively. This test took place over a 48-hour period and the sub-lethal effect, immobilization, was the single end point. In addition, LC50 for the marine bacterium Vibrio fischeri by smoked cigarette butts, smoked cigarette filters, and unsmoked cigarette tobacco was 0.6, 1.25, and greater than 970 cigarette butts/L, respectively. This study of V. fischeri took place over a 30-minute period and the sub-lethal effect, bioluminescence, was the single endpoint.

Micevska et al. [37] followed USEPA [40] protocols to perform daphnid bioassays and New South Wales Environmental Protection Agency [41] protocols for bacterium bioassays. Smoked cigarette butt leachates from 19 different brands of smoked cigarette butts were found to be toxic to Ceriodaphnia dubia at concentrations between 8.9 and 25.9 mg butts/L (48-hour EC50 (immobilization) and to Vibrio fischeri at concentrations between 104 and 832 mg butts/L (30-minute EC50 [bioluminescence]). This study also completed a Toxicity Identification Evaluation (TIE) phase I and preliminary phase II tests using USEPA [40, 42, 43] protocols. These evaluations identified nicotine and ethyl phenol as the most likely causative toxicants in cigarette butt leachate. However, the concentrations of these chemicals in the leachates were not measured.

Using the USEPA standard acute fish bioassay, Slaughter et al. [17••] analyzed cigarette butt-derived leachates for aquatic toxicity to saltwater and fresh water test fish. Survival was the single endpoint, and data were analyzed to identify the LC50 of machine-smoked cigarette butt leachates in the laboratory environment. The LC50 for leachate from smoked cigarette butts (with remnant tobacco intact) was approximately 1.1 cigarette butts/L for both the marine Pacific topsmelt (Atherinops affinis) and the freshwater fathead minnow (Pimephales promelas). Leachate from smoked cigarette filters without tobacco remnants was less toxic than that from smoked cigarettes with tobacco remnants, with LC50 values of 4.1 and 5.5 cigarette butts/L, respectively for both fish species. Unsmoked cigarette filters (without any tobacco remnants) were also found to be toxic, with LC50 values of 5.1 and 13.5 cigarette butts/L, respectively for both fish species. Toxicity was found to be highest for smoked cigarettes with remnant tobacco, but also for only the smoked filter (without tobacco) and to a lesser extent for the unsmoked filter.

In summary, cigarettes and their waste, deposited as discarded filters with remnant tobacco, contain many chemicals that may be harmful to the environment. These chemicals are sourced from agricultural treatments of tobacco plants, uptake from contaminated soils, additives instilled in the manufacturing process, the attached cellulose acetate filter, and combustion products generated in the course of smoking cigarettes. Limited studies of toxicity from these products to aquatic organisms have been reported, but given the total global burden of TPW, additional research is needed to explore the actual risks that this toxic waste has on freshwater and marine environments, the fate of such chemicals in aquatic environments, as well as their potential for bioaccumulation and human health effects.

## The Filter Farce

The discarded cigarette butt consists of unsmoked remnant tobacco, the paper wrap remnants, and the filter (99 % of cigarettes sold in the United States are filtered). Each of these components presents an individual environmental concern. In fact, as discussed above, the cigarette filter may compound the potential environmental effect of chemicals leached from butts because it is essentially a nonbiodegradable plastic collection of cellulose acetate fibers. Most filters have 2 layers of paper and/or rayon wrapping, the porosity of which acts to control the amount of airflow (ventilation) through the filter. Cigarettes also contain glues to hold the paper and filter together and alkali metal salts of organic acids (eg, sodium acetate) to maintain burning [44]. Although exposure to UV rays may eventually cause the filter to deteriorate into small pieces, the plastic particles and their toxicants may never disappear from water or soil and may continue leaching chemicals for up to 10 years [45, 46].

Cigarette manufacturers have promoted light and low-tar cigarettes that imply a health claim for these filtered (or ‘safer’) cigarettes. However, smokers who switched to low-yield, filtered brands in the 1950s and 1960s did not benefit from reduced exposures to tar and nicotine because of changes in their puffing behavior (known as ‘compensatory smoking,’) and design changes in manufactured cigarettes [47]. In the early 2000s, tobacco control researchers reported on how filter ventilation represents a dangerous, defective technology that could be regulated out of the cigarette market [48, 49].

The National Cancer Institute’s comprehensive review of light and low-tar cigarettes [50] concluded that “Epidemiological and other scientific evidence, including patterns of mortality from smoking-caused diseases, does not indicate a benefit to public health from changes in cigarette design and manufacturing over the last 50 years.” Under the 2009 U.S. Family Smoking Prevention and Tobacco Control Act [51] tobacco companies are now prohibited in the United States from the advertising or labeling of tobacco products with the descriptors “light,” “mild,” or “low”. These terms have misled smokers about implied benefits of filtered cigarettes since their market entry, and, thus, claims about filters that reduce yield of tar have been found to be misleading and fraudulent [52]. The large scale uptake of filtered cigarettes may have been associated with a reported histologic shift in predominant lung cancer type from squamous cell to adenocarcinoma [53, 54].

Smokers may be discouraged from quitting as many still believe that filtered cigarettes protect their health, and young people may find it easier to inhale their first puff with filtered cigarettes. Because of these issues, filters may be considered as defective products in terms of protecting smokers’ health. Because of their relative nonbiodegradability and the preliminary research indicating the toxicity of TPW to a variety of aquatic organisms, the filter tip as product source of environmental contamination may be a target for product alteration under the principle of Extended Producer Responsibility (EPR see Section 2, below).

## Conclusions and Recommendations

TPW is ubiquitous, environmentally hazardous, and significant community nuisance. Although anti-littering laws exist that may apply to TPW in many jurisdictions, most enforcement is directed at large littering problems such as illegal dumping. Enforcement of such laws directed toward individual smokers’ TPW littering is impractical and has been clearly ineffective in preventing the accumulation of TPW. Research on both the extent and nature of the TPW problem, the potential chemical impact on the environment, wildlife, and humans, the defectiveness of filtered cigarettes, and the tobacco industry’s efforts in avoiding responsibility for TPW environmental contamination is needed. The findings would strengthen the evidence base for taking action on this global environmental problem.

TPW mitigation requires novel environmental interventions and new partnerships between tobacco control and environmental groups. Many of these interventions would serve to reduce the social acceptability of smoking while reducing the environmental burden of TPW. Based on this review of the TPW problem, the following policy approaches are suggested:

1. Increase public awareness about the toxicity and other environmental impacts of TPW
  Environmental advocacy joined with tobacco control advocacy can be an effective approach to the TPW issues. In fact, the tobacco industry has ‘feared’ such an alliance among these different camps, and has sought to invest in environmental advocacy that emphasizes TPW cleanups, hand-held ashtrays, butt receptacle installations, and other downstream approaches [13]. Mobilizing public opinion on exposure to second hand smoke has resulted in myriad local and state regulations to prevent this environmental health hazard (See: http://www.no-smoke.org/goingsmokefree.php?id=519). Thus, similar advocacy, with mobilization of environmental groups, will be necessary to implement effective policies to prevent and mitigate the environmental burden of TPW.
2. Apply the Extended Producer Responsibility Principle to TPW
  EPR requires total life cycle environmental improvements, placing liability, economic/financial, physical, and informational responsibilities onto the manufacturers of the waste product [55]. Product stewardship (PS) overlaps principles of EPR but extends responsibility to all parties involved in the life cycle of the product. In the case of TPW, this would include sellers, distributors, and perhaps even facilitators such as bars and restaurants that allow outdoor smoking on their premises. A key focus of both EPR and PS involves postconsumer take-back and final disposal. This could involve a deposit-return scheme or simply require manufacturers to take back all discarded TPW. EPR has been emphasized in Europe since the early 1990’s, and it was incorporated into official European Union environmental policy in 2002. However, EPR regulations have not yet been considered at the Federal level in the United States [56]. Nevertheless, as of October 2010, 32 US States have enacted EPR laws that mandate costs of recycling or safe disposal of consumer products to be covered by the manufacturers of these products; these products include batteries, carpets, cell phones, other electronics, fluorescent lighting, mercury-containing thermostats, paint, and pesticide containers [57].
3. Apply the ‘Precautionary Principle’ to TPW
  This principle implies that it is not necessary to have identified each and every TPW toxic chemical and its potential health effects before regulating TPW and is a hallmark of environmental health policy in the United States and elsewhere. Such policies re-focus the concern on TPW “upstream” from the consumer, community, and environment to the manufacturers and distributors of tobacco products.
4. Label Cigarette Packages
  With evidence for the effectiveness of cigarette package warning labels [58], additional package labels and public information about the toxicity of discarded butts may be considered. These would include specific instructions for the safe disposal of the toxic waste product and brief information about why this disposal is important. These labels would contribute to public information about TPW toxicity.
5. Deposit/return Schemes
  As for deposit schemes, Oregon and several other U.S. states have implemented deposit-return schemes on glass and metal beverage containers as a way to reduce the environmental burden of discarded beverage containers. These laws impose a consumer-paid monetary deposit on specified items that is reimbursed when the item is returned. The Oregon law reduced litter and increased recycling, with return rates of up to 90 % and reduction of roadside beverage container litter from 40 % to <6 % of total litter [59]. Similarly, cigarettes could be sold with a “butt deposit” to be refunded when the butts are returned to the vender or perhaps to a hazardous waste disposal facility. This could encourage smokers to behave more responsibly and could provide income to butt retrievers. It would also increase the costs of smoking, thus having a beneficial effect on cigarette consumption. Further, vendor reluctance to accept returned butts (due to aesthetic, logistical, or storage problems) might reduce the number of outlets selling cigarettes. Recycling schemes for TPW have been proposed by a variety of environmental groups and commercial entities, including those funded by the tobacco industry [60, 61].
6. Cost Recovery
  Tobacco litter abatement costs to cities are substantial, even when the costs of potential environmental toxicity and potential effects on tourism are excluded [62]. One solution to reducing toxic waste from computers, telephones, and televisions is a consumer-funded Advanced Recycling Fee (ARF); this is assessed at the time of purchase for these products and it is meant to pay for the costs of recycling and disposing properly of any non-recyclable material; California and Maine have implemented such fees on electronics [63].
  Total public litter abatement costs to a city range from $3 to $16 million [62]. TPW comprises 23 %–36 % of all visible litter, and, thus, the costs borne by the public for TPW range from $1 to $5 million for a typical city. The costs of mitigating this externality of TPW in a mid-sized metropolitan area (such as has been implemented in San Francisco) can be offset by a fee of approximately $0.20–$0.40 per pack. These fees would then increase the cost of cigarettes, thereby reducing consumption.
7. Litigation
  Litigation brought by States against the tobacco industry has focused mainly on recovering the State-funded health care costs attributable to smoking. As for environmental costs, the tobacco industry could be held responsible for cleanup and nuisance costs associated with tobacco products. EPR may then be invoked to address tobacco industry responsibility. Under this principle, litigation has been pursued against manufacturers of several other products that have damaged the environment through class action lawsuits. These suits are typically based on 2 legal theories: negligence and nuisance. The primary basis for a negligence case would be proof of the defendant’s wrongful conduct in failing to prevent environmental damages from normal usage of their products (again, invoking the ‘precautionary principle’) [13]. Nuisance-based lawsuits may invoke the “right of quiet enjoyment” that is disrupted such that a tort is being committed. Litigation against the tobacco industry by State or local entities may be considered as a means to recover environmental cleanup and nuisance costs.
8. Product Changes
  Some hazardous products have been banned entirely by State and local authorities through restrictions on sales and distribution. These include pop-tops on aluminum cans, plastic tampon applicators, and non-fire-safe children’s clothing [13]. Thus, States could consider banning the sale of filtered cigarettes if these were to be considered an environmental hazard and nuisance burden. (In fact, a bill has been submitted in 2014 to the California Legislature to ban the sale of single-use filtered cigarettes for environmental reasons [64].)
  There may in fact be significant positive behavioral and health impacts if the sale of filtered cigarettes were prohibited because such prohibition may reduce consumption of cigarettes in general or smoking initiation among children by making the cigarette less palatable. Filters are a marketing tool and not a health device, and, thus, banning them on environmental grounds may make sense, both as an environmental intervention and as a public health intervention.
  The issue of whether there is a safe cigarette for consumers has been laid to rest, and the environmental burden of TPW will benefit from the absence of the defective cellulose acetate filter. However, one may wonder whether the Family Smoking Prevention and Tobacco Control Act signed into law in 2009 would preempt State or local actions to ban the sale of filtered cigarettes. This legislation in fact preserves the rights of states to raise tobacco tax rates, implement and enforce comprehensive smoke-free laws, adequately fund strong state tobacco prevention programs, enhance access to smoking cessation, and *take any actions to restrict the sale and distribution of tobacco products* [65]. Thus, banning the sales of filtered cigarettes may be considered by States as a means of significantly reducing the TPW environmental and economic burden at the State or local level.
